# Cerebral Protection in Pediatric Cardiac Surgery

**DOI:** 10.1007/s00246-024-03748-7

**Published:** 2025-01-08

**Authors:** Sheida Shams-Molkara, Vitor Mendes, François Verdy, Maria-Helena Perez, Stefano Di Bernardo, Matthias Kirsch, Amir-Reza Hosseinpour

**Affiliations:** 1 Faculty of Biology and Medicine, University of Lausanne, Lausanne, Switzerland; 2 Perfusion Unit, Service of Cardiac Surgery, Geneva University Hospital, Geneva, Switzerland; 3 Perfusion Unit, Service of Cardiac Surgery, Lausanne University Hospital, Lausanne, Switzerland; 4 Pediatric Intensive Care Unit, Service of Pediatrics, Department of Women-Mother-Child, Lausanne University Hospital and University of Lausanne, Lausanne, Switzerland; 5 Pediatric Cardiology Unit, Service of Pediatrics, Department of Women-Mother-Child, Lausanne University Hospital and University of Lausanne, Lausanne, Switzerland; 6 Service of Cardiac Surgery, Lausanne University Hospital and University of Lausanne, Rue du Bugnon 46, 1011 Lausanne, Switzerland

**Keywords:** Cerebral protection, Cerebral protection in cardiac surgery, Pediatric cardiac surgery, Complications of cardiac surgery

## Abstract

Cardiac surgery, both adult and pediatric, has developed very rapidly and impressively over the past 7 decades. Pediatric cardiac surgery, in particular, has revolutionized the management of babies born with congenital heart disease such that now most patients reach adult life and lead comfortable lives. However, these patients are at risk of cerebral lesions, which may be due to perioperative factors, such as side effects of cardiopulmonary bypass and/or anesthesia, and non-perioperative factors such as chromosomal anomalies (common in children with congenital heart disease), the timing of surgery, number of days on the intensive care unit, length of hospitalization and other hospitalizations in the first year of life. The risk of cerebral lesions is particularly relevant to pediatric cardiac surgery given that cerebral metabolism is about 30% higher in neonates, infants and young children compared to adults, which renders their brain more susceptible to ischemic/hypoxic injury. This issue has been a major concern throughout the history of cardiac surgery such that many preventive measures have been implemented over the years. These measures, however, have had only a modest impact and cerebral lesions continue to be a major concern. This is the subject of this review article, which aims to outline these protective measures, offer possible explanations of why these have not resolved the issue, and suggest possible actions that ought to be taken now.

## Introduction

Cardiac surgery, both adult and pediatric, has developed very rapidly and impressively over the past 7 decades. Pediatric cardiac surgery, in particular, has revolutionized the management of babies born with congenital heart disease such that now most patients reach adult life and lead comfortable lives. However, these patients are at risk of cerebral lesions, which may be due to perioperative factors, such as complications of cardiopulmonary bypass and/or anesthesia, and non-perioperative factors such as chromosomal anomalies (common in children with congenital heart disease), timing of surgery, number of days on the intensive care unit, length of hospitalization and other hospitalizations in the first year of life. Complications of cardiopulmonary bypass are particularly noticeable, and considerable work has been done to minimize these.

Most cardiac operations require cardiopulmonary bypass, which was developed by John Gibbon Jr in Philadelphia and used in May 1953 for the first time [1]. This was a major development and the real point of take-off of cardiac surgery. Before this, cardiac surgery was very limited and done in one of two ways [2]. One way, led by John Kirklin, was using surface cooling allowing deep hypothermic circulatory arrest, based on experimental studies on the protective effects of hypothermia by Bigelow in Toronto in the 1940s [3]. The other way, led by Walton Lillehei, was by cross-circulation using patients’ parents as living cardiopulmonary bypass machines, but this was stopped because of brain damage caused in one patient’s parent. Clearly, a bypass machine was a much more effective and practical solution but was very soon found to have neurologic complications in the form of memory disorders, visual field defects, aphasia, alexia, agraphia, cognitive disorders, seizures and hemiplegia. These complications were first reported in 1954 in adults after mitral valve surgery but were subsequently also reported in other cardiac operations [4–6].

## The Incidence of Neurologic Lesions

The incidence varies greatly depending on how detailed your neurologic assessment is, i.e. how hard you look for neurologic injury. Often, the assessment is confined to the fact that patients wake up, open their eyes, move all limbs and do not have seizures. Such patients are often declared as free of neurologic lesions, ignoring the fact that the brain has many other functions. This applies particularly to neonates, infants and even children, who are not routinely assessed beyond this. Older patients may receive a more detailed assessment in the form of clinical neurologic examination. An even more detailed assessment would include tests of memory, speech, reading and cognitive disorders. A thorough assessment, however, would include detailed psychomotor tests. Depending on the level of detail of neurologic assessment, the incidence of neurologic lesions was between 2% and 50% of all cardiac operations in the early years of cardiopulmonary bypass [4–6]. This raises the question of what the mechanisms of these lesions are.

## The Mechanisms of these Cerebral Lesions

Several possibilities have been debated over the years. One possibility is embolism, which could be of air or thrombi. This has always been at the top of the list of possible explanations for cerebral lesions given the complex structure of the bypass circuit consisting of tubes, connections, taps and membranes, thus providing many opportunities for thrombosis and air entry even in the absence of any accident in the conduct of the procedure. Another possibility may be periods of hypoperfusion/hypoxia as the cardiovascular system is being manipulated in drastically unphysiological manners. This applies to many aspects of cardiovascular physiology, but one that is particularly relevant is the autoregulation of cerebral blood flow; we do not know what happens to this process on cardiopulmonary bypass [7]. Yet another possibility is that these lesions may be consequences of the inflammation and edema that are well-known consequences of cardiopulmonary bypass due to contact of blood with foreign material, which activates the complement system and therefore inflammation. All these possible mechanisms have been targeted over the years in many investigations and attempts to neutralize them. These investigations and attempts may be classified into 2 groups:

- Those focusing on improving the hardware of the bypass circuit
- Those focusing on improving the techniques of conduct of bypass

## The Hardware

### The Original Machines

Originally, bypass machines were very large and there was only one size for all patients. This had two undesirable consequences. First, this meant that blood had contact with much more extensive foreign surfaces and, therefore, with more risk of formation of thrombi and embolism and possibly stronger inflammatory response. Second, priming volumes were very large (measured in liters). The priming volume is the volume of fluid required to fill the entire circuit before connecting this to the patient. Such ‘priming’ of the circuit is necessary because the circuit merges and becomes continuous with the patient’s circulation and must, therefore, contain no air at all. Such large priming volumes, however, meant that the priming fluid had to include blood, otherwise the patient would be dangerously hemodiluted. In pediatric cardiac surgery, given the small size of patients, this amounted to a massive blood transfusion with all the side effects and complications that accompany this.

The large size of the bypass circuits, however, was not the only problem in the original machines; an additional source of complications was that gas exchange was done with bubble oxygenators. These worked by bubbling gases with varying concentrations of oxygen directly into the blood within the container of the oxygenator device. This was quite traumatic to red blood cells because of surface tension created by being in direct contact with the gases, thus causing hemolysis and potentiating the inflammatory reaction to cardiopulmonary bypass. In addition, this was associated with a significant risk of air embolism.

These original bypass machines have been refined in many ways over the years. These developments have led gradually to the modern machines that are in use today.

### Modern Machines

Modern bypass machines have more components, and each component is available in different sizes. In fact, all components have become increasingly miniaturized. Consequently, bypass circuits are much better matched to the size of patients, and priming volumes have become accordingly much smaller [8]. Also, bubble oxygenators became obsolete in the late 1980s and were replaced with membrane oxygenators, which do not involve direct blood-gas contact, thus reducing the risk of air embolism and trauma to red blood cells [9]. In addition, the circuits have been fitted with arterial and venous filters as well as various monitoring devices [10]. Filters were quite challenging to develop, and this took a long time because these devices, although meant to remove thrombi, can also produce thrombi by themselves, both downstream and upstream.

## Techniques of Conduct of Cardiopulmonary Bypass

The conduct of cardiopulmonary bypass has evolved continuously over the years with multiple adjustments being introduced, some concomitantly and some at different times. These are summarized here:

### Increasing the Technical Rigor of Bypass

Surgeons became more mindful of cannulation sites and angles, aiming to optimize cerebral blood flow. In adults, surgeons began to look for and avoid atheromatous and calcific plaques when cannulating the aorta to minimize the risk of these lesions becoming detached from the aortic wall and embolizing to vital organs [11]. This concern also led to the manufacture of less traumatic clamps for the aorta and, at the same time, the desire to avoid clamping the aorta altogether when possible. Surgeons also became more mindful of rigorous de-airing of the heart at the end of the procedure and stricter monitoring of anticoagulation. In adults, the process of de-airing the heart has been supplemented with the insufflation of carbon dioxide (CO_2_) into the operative field; this strategy aims to displace air to minimize its entry into the heart, replacing it with CO_2_, which is much more soluble and, therefore, much less likely to turn into bubbles. This is used much less in pediatric cardiac surgery because it is somewhat cumbersome given the much smaller size of patients.

### Reducing Priming Volumes

Although priming volumes were reduced drastically in parallel with the miniaturization of the circuits in babies, these were reduced even more by strategically placing the bypass machine as close to patients as possible such that the tubes and cannulas could be shortened maximally, thus further reducing the use of blood products.

### Less Hemodilution

A degree of hemodilution is very useful for cardiopulmonary bypass because it decreases the viscosity of blood, and therefore less mechanical force/pressure is needed from the bypass pump to maintain adequate blood flow with less risk and amount of hemolysis and its undesirable consequences on the entire body, including and especially the brain. In addition, hemodilution allows the procedure to be done with less blood transfusion, which is very beneficial given the possible complications of transfusion including a systemic inflammatory response. However, this must be balanced against the risk of ischemic insult to the tissues. Originally, such hemodilution was done excessively, with target hematocrits in the order of 20% or even less [12]. This was changed in the late 1990s thanks to experimental data from Boston showing that a hematocrit of about 30% reduces blood viscosity sufficiently for adequate perfusion and that babies’ neurological development is better at this level of hemodilution [13–16]. This became the norm.

### Hypothermia

Hypothermia has been known to protect tissues against ischemia/hypoxia since before the advent of cardiopulmonary bypass, initially based on the experimental work of Bigelow in the 1940s in Philadelphia doing surface cooling of dogs showing that cerebral metabolism and oxygen consumption are reduced in such conditions, although later this has also been shown in humans [3, 17]. This allows cessation of the circulation for longer than it is possible at normothermia without causing cerebral damage. Circulatory arrest is not tolerated in normothermia for more than about 3–4 min, after which irreversible cerebral lesions occur. However, both clinical and experimental work have shown that lowering body temperature by 10 ℃ results in a four-fold decrease in cerebral metabolic activity, thus protecting the brain against ischemia [18, 19]. Such protection allowed surgeons to carry out some limited cardiac surgery prior to the advent of cardiopulmonary bypass by surface cooling and circulatory arrest, but this technique became obsolete once bypass machines became available given than cerebral perfusion was secured by the machine. Nevertheless, a degree of hypothermia continued to be used during bypass as additional protection, especially given that it was not yet clear whether the early bypass machines could be trusted not to break down during the procedure. Subsequently, hypothermia gradually became incorporated in cardiac surgery and, by the 1970s, it became an important component of pediatric cardiac surgery [20–26].

Although cardiopulmonary bypass allowed the circulation to be maintained during cardiac operations, this was not always desirable; in some cases, blood flow had to be stopped completely to allow an adequate correction such as in surgery on the aortic arch. In such cases, further cooling was implemented, down to deep hypothermia, with the main objective of protecting the brain, before arresting the circulation (so-called *Deep Hypothermic Circulatory Arrest* [DHCA]). This was, and still is, supplemented with the placement of ice on the head during the circulatory arrest period to minimize passive rewarming of the brain [24, 27]. Hypothermia, however, needed to be defined in terms of actual temperatures. This has been done by consensus of experts as mild (28–34 ℃), moderate (20–28 ℃), deep (14–20 ℃) and profound (< 14 ℃) [11, 28].

Initially, in the 1960s and early 1970s, hypothermia was achieved by surface cooling before going on bypass to reduce the time spent on bypass [20, 21]. This was changed in the late 1970s and early 1980s, by Aldo Castañeda in Boston and Paul Ebert in San Francisco, to cooling and rewarming on bypass using a heat exchanger, pointing out that this results in more efficient and uniform body temperature changes [22, 29]. This was done, and still is, by vasodilating the patient and increasing the pump flow to accelerate the cooling and rewarming processes so that the duration of the cardiopulmonary bypass would not be excessively prolonged. Nevertheless, these changes in temperature must not occur too fast either so that the changes in body temperature may indeed be uniform and no bubbles form in the blood during rewarming. In practice, this means maintaining a difference of no more than 10 ℃ between the temperatures of the venous and arterial blood in the circuit during cooling and rewarming [23, 25]. Given the additional risk of bubble formation in the blood during rewarming, however, it is recommended that rewarming should take twice as long as the cooling period as an added safety measure [23, 26]. In addition, the Boston group showed that post-rewarming hyperthermia should be avoided as this exacerbates cerebral lesions [30, 31].

Note that, although hypothermia affects the entire body, it aims primarily at cooling the brain given that cerebral protection is the primary objective of this strategy. Therefore, the temperature that needs to be monitored during this process is cerebral temperature. This is best done by monitoring nasopharyngeal or esophageal temperatures as these correlate well with cerebral temperature; correlation with rectal and vesical temperature is less strong [23].

### More Careful Management of Carbon Dioxide (CO_2_) and Acid–Base Balance During Hypothermia

Hypothermia, by decreasing the metabolism of the entire body, decreases CO_2_ production such that patients become increasingly alkalotic. Although the reduction in metabolism protects the tissues, the accompanying alkalosis has two undesirable effects. First, it causes systemic vasoconstriction, which includes cerebral vasoconstriction, thus putting the brain in danger of hypoperfusion [32–35]. Second, it shifts the oxygen dissociation curve, which is already shifted to the left due to hypothermia, further to the left, thus decreasing oxygen delivery to the tissues (Bohr effect).

This issue was addressed in the early 1990s, both experimentally and clinically, when it became clear that such alkalosis must be counteracted [36–40]. This is done by not allowing the partial pressure of carbon dioxide (pCO_2_) to decrease, which may be achieved by decreasing CO_2_ clearance through the gas-exchange membrane of the bypass circuit, thus allowing the CO_2_ that is naturally produced by the patient (even though CO_2_ production is diminished) to accumulate in a controlled fashion. If this proves insufficient, CO_2_ may be added directly into the patient’s blood through the gas exchange membrane. This strategy is known as pH–stat, i.e. adding CO_2_ to keep pH constant. It secures cerebral perfusion and oxygenation.

The pH–stat strategy is often contrasted with the so-called alpha-stat strategy, which is another approach to acid–base management on cardiopulmonary bypass [40, 41]. This contrast, however, is somewhat of an exaggeration of facts and may therefore be misleading; it deserves to be clarified.

Alpha-stat means keeping alpha constant. Alpha refers to the alpha-imidazole ring of histidine, which is an important intracellular buffer. Therefore, alpha-stat means maintaining this intracellular buffer, thus maintaining the net charge on intracellular proteins constant, which allows these to function optimally despite temperature changes and the fact that pCO_2_ decreases in hypothermia. The alpha-stat strategy relies on the natural physiological adaptation to hypothermia without artificially altering pCO_2_ levels, respecting the natural reduction in pCO_2_ as part of the response to hypothermia. However, the fact remains that reduction in pCO_2_, with accompanying alkalosis, puts the brain in danger. Therefore, pCO_2_ must be maintained within a safe range, and this is done in a similar manner to pH–stat described above. This is to say that, essentially, the actions required to prevent harmful drops in pCO_2_ are similar whether you adhere to alpha-stat or pH–stat. Nevertheless, some people argue that the actions that must be taken are not exactly the same in these two strategies in the sense that alpha-stat accepts a pCO_2_ at the lower end of the normal range whereas pH–stat aims at the middle or upper end of the range, i.e. pH–stat is more aggressive with the maintenance of pCO_2_. This argument, however, is very weak given how narrow the normal range of pCO_2_ is. In reality, the difference between these two strategies is more conceptual and philosophical than practical. Indeed, in conceptual and philosophical terms, it may be said that alpha-stat aims to maintain intracellular pH constant whereas pH–stat aims to maintain blood pH constant at different temperatures. In practical terms, however, whether you call it alpha-stat or pH–stat, pCO_2_ must not be allowed to drop as this puts the brain in danger.

### Addition of Selective Cerebral Perfusion During Circulatory Arrest

Hypothermia prolongs the tolerable period of circulatory arrest significantly, but this period remains limited such that every effort must be made to keep the procedure short and introduce periods of intermittent reperfusion if possible [31, 42, 43]. In addition, the exact tolerable period is not certain, although it is said to be between 30 and 40 min in deep hypothermia, beyond which the risk of neurological lesions increases substantially [17, 23]. Nevertheless, some rules of thumb have been proposed over the years based on opinions rather than hard science. For example, one such practical rule of thumb states that 3 min of circulatory arrest is tolerable at normothermia, and then the tolerable time is doubled for every 5 ℃ reduction in temperature: 6 min at 32 ℃, increasing to 12 min at 27 ℃, and to 24 min at 22 ℃ and 48 min at 17 ℃. However, this is a gross over-simplification such that these periods do not guarantee safety. Also, the safe period may well be variable between patients. Clearly, deep hypothermia alone is unlikely to fully protect the brain during the periods necessary for many surgical procedures, as can be seen in the sobering results that have been published from the 1980s onwards, specifically the high incidence of seizures, cognitive disorders and lesions seen on magnetic resonance imaging [31, 44, 45]. This led to the introduction of selective cerebral perfusion as an adjunct during circulatory arrest, thus providing some cerebral perfusion while the rest of the body is in circulatory arrest [46–48].

Selective cerebral perfusion may be done in various ways, but typically it involves advancing the aortic cannula into the brachiocephalic artery, which is then snared, thus perfusing the brain through the right carotid and right vertebral arteries, relying on the circle of Willis to ensure perfusion of the entire brain. The circle of Willis, however, is not always complete, which puts the left cerebral hemisphere in danger of hypoperfusion. For this reason, cerebral perfusion is occasionally done bilaterally with an additional cannula placed in the left carotid artery. But this is generally too cumbersome such that most surgeons do not do this. Alternatively, cerebral perfusion during circulatory arrest may be done retrogradely through the superior vena cava, which may potentially be safer as this does not rely on the circle of Willis [23, 49–51]. Either way, cerebral perfusion may also allow the procedure to be done at higher temperatures, avoiding deep hypothermia, thus reducing cooling and re-warming times and therefore also bypass times and their associated possible complications [52].

Although the use of selective cerebral perfusion makes sense intuitively, it is not yet supported with hard science and the published outcomes are variable [23]. In the Boston circulatory arrest trial, DHCA was compared with continuous bypass: there were neurologic differences in the form of higher perioperative incidence of seizures with DHCA compared to continuous bypass, but no difference 8 years later based on any neurocognitive test including intelligence [31]. Also, in a retrospective study from Philadelphia, the incidence of postoperative seizures in neonates following cardiac surgery was not different whether the operations were done under DHCA alone or with regional cerebral perfusion [53].

Overall, the strategy of selective cerebral perfusion does raise several questions that are yet to be answered and are summarized here [43, 54]:

1. Should this be done in antegrade or retrograde fashion?
2. If antegrade, is unilateral perfusion sufficient, or we must perfuse bilaterally despite this being somewhat cumbersome?
3. Cerebral perfusion should be done at what temperature, what flow and what pressure? At present, these three criteria vary significantly between different surgical teams.
4. Are we relying too much on it?
5. How can we guard against its complications such as damage to the cannulated vessels, embolism and intracerebral bleeding?
6. Is selective cerebral perfusion better than DHCA alone if the period of circulatory arrest is not too long? So far, no difference has been convincingly demonstrated between these two strategies [23, 31, 53, 55].
7. Given the above-mentioned doubts and risks, should we use simply DHCA without selective cerebral perfusion if the duration of the procedure is not expected to be too long? This would avoid additional cannulas and trauma to small vessels, make the operation easier and faster, and reduces exposure to cardiopulmonary bypass. However, this is difficult to gauge given that there is no absolute safe time, and this strategy may become problematic if the procedure turns out to take longer than expected, with the risk of permanent neurologic injury that is time-dependent [56, 57]. Nevertheless, in very small babies (e.g. < 2 kg), DHCA without cerebral perfusion may be preferable to avoid injuries to vessels and intracerebral hemorrhage, but this requires trials for confirmation.

### Reducing the Amounts of Circulating Cytokines

The inflammatory state caused by cardiopulmonary bypass, with its accompanying high amounts of circulating cytokines, puts all organs at risk, including the brain. This has led to the development of two techniques to remove as much of these cytokines as possible, namely ultrafiltration and hemadsorption.

Ultrafiltration works by diffusion of substances across a membrane within an air-tight chamber, just like hemodialysis [58]. The device is fitted onto the bypass circuit and may be used continuously during the procedure (so-called conventional ultrafiltration—CUF), or after the termination of the bypass as if it were a hemofiltration device (so-called modified ultrafiltration—MUF) or both. It is usually conducted to remove as much fluid as patients’ cardiovascular stability allows, which, in practice, means achieving zero balance if possible. In doing so, it consistently reduces the circulating amounts of cytokines and improves postoperative outcomes by reducing fluid overload, reducing the need for blood transfusion, and improving both myocardial and pulmonary functions. Consequently, it is assumed that this also improves cerebral outcome given that it seems beneficial to other organs, but this has not been specifically demonstrated yet.

Conversely, hemadsorption works by passing blood through a cartridge containing sorbent material that removes solutes by direct binding through hydrophobic interactions, ionic attraction, hydrogen bonding or van der Waals forces (weak electrostatic forces that attract neutral molecules to one another) [59]. This was first developed as an extracorporeal blood purification strategy for removing inflammatory mediators and bacterial toxins from the blood of patients with sepsis but has also been used for various other conditions including in the treatment of acute poisoning, drug overdose, chemical exposure and for removal of toxins in various renal, hepatic and immunologic diseases. Its use in cardiac surgery (added to the bypass circuit), however, is recent and its benefit is yet to be demonstrated. It differs from ultrafiltration in that ultrafiltration works by diffusion across a membrane whereas this works by direct binding onto sorbents.

## Additional Measures for Cerebral Protection

Many measures for cerebral protection have been added to the management of patients undergoing surgery with cardiopulmonary bypass [23, 43]. These include tight control of blood glucose, the use of agents that may reduce cerebral metabolism, and other agents that reduce the inflammatory response to cardiopulmonary bypass, edema and intracranial pressure.

Tight control of blood glucose is important because hyperglycemia augments intracellular acidosis, which harms neurons [60, 61]. Hyperglycemia may occur easily during cardiac surgery due to 3 factors, namely the stress response to surgery, hypothermia and the administration of corticosteroids (see below).

Cerebral metabolism may be reduced by certain anesthetic agents, although the exact choice is still a matter of debate since the results of studies are variable [23, 43, 62–66]. Notably, barbiturates, especially thiopental, are known to reduce cerebral metabolism, but these are impractical by being long-acting and being negative inotropes. Thiopental has largely been superseded by propofol, which is short-acting and also reduces cerebral metabolism [62]. Some volatile anesthetic agents are also neuroprotective, but their solubility increases at low temperatures, which slows down their rate of reaching adequate levels in the central nervous system during cooling, and too high levels during re-warming resulting in too deep levels of anesthesia [63].

Reduction of the inflammatory response to cardiopulmonary bypass, oedema, brain swelling, and intracranial pressure are addressed with the administration of corticosteroids and mannitol, although other agents are also being studied such as allopurinol, erythropoietin and dexmedetomidine [67–69].

## Monitoring

Adequate cerebral protection requires adequate monitoring [13]. This includes the monitoring of all the above-mentioned measures that have been implemented to protect the brain, such as the hemodynamic parameters of cardiopulmonary bypass, temperature, hematocrit, acid–base balance and blood glucose. However, other monitoring modalities have also been developed specifically targeting the brain from various points of view:

1. Cerebral blood supply and detection of microemboli, using transcranial doppler ultrasonography [70].
2. Cerebral oxygenation, using near-infrared spectroscopy (NIRS) [61–74]. This is the most popular method given its simplicity. This has been employed since the mid-1970s as a non-invasive continuous real-time measure of the balance between cerebral oxygen delivery and consumption. It has been used in a variety of surgical specialties including neurosurgery, trauma, vascular and cardiac surgery. It is also used in intensive care units. Essentially, it measures the overall saturation of the cerebral frontal cortex. It is particularly useful in cardiac surgery by providing a non-invasive real-time measure of local tissue perfusion while on cardiopulmonary bypass, thus indicating if and when interventions may be needed to avoid cerebral hypoxia. This technology is similarly used on the lower body to monitor splanchnic perfusion.
3. Cerebral activity and metabolism, by measuring the jugular venous bulb oxygen saturation using fiberoptic technology [75]. A low reading indicates increased cerebral metabolism.
4. Cerebral electrical activity, using electroencephalography, bispectral indices and somatosensory evoked potentials, all of which are blunted in hypothermia indicating adequate cooling of the brain [23, 76, 77].

It must be said, however, that none of these neuromonitoring modalities has been shown to lead to better neurologic outcomes yet.

## What are the Results of these Efforts?

Overall, there is no doubt that cardiac surgery has progressed rapidly and impressively over the past 7 decades such that the incidence of major complications has markedly decreased as the hardware and techniques have been refined. However, specifically in terms of neurologic outcomes, progress has been at best modest, despite all the above-mentioned strategies that have been developed to protect the brain. Indeed, many strategies have emerged, but these are generally ‘home-grown’ (center-specific) and unsupported with hard science, except for avoiding excessive hemodilution, which is well supported. As is usually the case in medicine, and in other walks of life, when there are several ways of trying to achieve the same objective, it means that none of them are perfect; the perfect way (if figured out) would eliminate all other ways. This imperfection is reflected in the sobering neurologic outcomes reported in both adult and pediatric cardiac surgery:

1. In adults, neuropsychological deficits, including cognitive dysfunction, occur in 30–60% after surgery with cardiopulmonary bypass [78–82].
2. In pediatric cardiac surgery, the incidence of neurologic complications varies between 2% and 50%, again depending on the criteria and level of details used for the diagnosis of such complications [78, 83–87].
3. The incidence of postoperative seizures remains significant in neonates [53, 56].
4. The incidence of Attention-Deficit Hyperactivity Disorder following pediatric cardiac surgery is significantly higher than that for normal children of the same age [84].
5. Nearly 50% of school-age survivors of neonatal cardiac surgery exhibit some neurobehavioral symptoms [86]. This could be in terms of IQ, attention deficits, impulse control, memory, speech motor skills or academic function.

## Why are We Failing to Resolve this Issue?

This remains a matter for debate and several possible answers have been proposed.

One possibility is that maybe our monitoring is inadequate in the sense that we detect problems too late when damage is already done.

Another possibility that is increasingly mentioned is that we have focused on intra-operative factors, but other factors may also be important such as chromosomal anomalies (common in children with congenital heart disease), timing of surgery, number of days on the intensive care unit, length of hospitalization, other hospitalizations in the first year of life, issues of fetal circulation and brain maturation as well as social factors. These may all be linked such that a malformation may affect the fetal circulation, which affects cerebral blood flow and oxygenation, which affects neuronal development causing a combination of white matter and neuronal/axonal disease. In other words, it is increasingly believed that cerebral lesions begin in utero and may be exacerbated later by cardiac surgery given that the brain of such babies would be particularly vulnerable to ischemia and hypoxia. This means that our operative protective measures come into play too late when the damage is already largely done [78, 87–91]. This idea is supported by the finding that the brain of fetuses with congenital heart disease grow less in the third trimester of pregnancy compared to healthy fetuses, particularly in those with abnormal aortic outflow, and this predicts poor further cerebral development [90, 91]. More specifically, 20% of neonates with critical congenital heart disease have preoperative white matter injury on magnetic resonance imaging, particularly in cases of impaired maternal–fetal environment (e.g. in the presence of gestational hypertension, diabetes or thyroid disorders) [88]. Also, the brain of the term infant with complex congenital heart disease resembles that of a 35-week premature neonate [87]. All these factors may well play important parts in the pathogenesis of cerebral lesions and status in babies with congenital heart disease, and the basis of the term e*ncephalopathy of congenital heart disease,* which was coined in 2014 by William Gaynor in Philadelphia [92].

Further data suggesting that the issue of cerebral lesions may be related to non-surgical factors was provided by a 16-years’ follow-up of the patients that had been originally studied in the Boston circulatory arrest trial [93]. These patients, who were adolescents by this stage, underwent both neuropsychological assessment and structural brain imaging. This study confirmed that, apart from slightly worse visual and spatial function amongst patients in the DHCA group, there was no long-term difference in outcome between DHCA and low-flow continuous bypass. However, a secondary finding of this study was that outcomes were affected by genetic factors, impaired maternal–fetal environment, family stability and social class.

Clearly, the probability of non-operative etiologies for cerebral lesions is strong. Nevertheless, operative factors remain important, as supported by the fact that cerebral problems are also seen after cardiac surgery in adults (as mentioned above), who were perfectly healthy for several decades prior to their operation, and whose brains are supposedly less vulnerable than that of neonates and infants. However, cerebral injuries may not necessarily be similar in adults compared to children, and they may not have been investigated similarly. Indeed, adults may be assessed much more thoroughly than infants and children, including with detailed psychomotor and cognitive tests, thus exposing issues that may otherwise remain hidden. Therefore, these two groups of patients may not be easily comparable.

## How Should We Proceed?

First and foremost, we must establish how best to follow these patients up and monitor their neurodevelopment so that we can assess and compare the impact of our various treatment strategies. Follow-up methods are currently very variable in different centers in terms of both techniques and depth of search for lesions. Ideally, such assessment should include not only thorough neurologic examination and imaging but also detailed psychomotor and cognitive tests. These tests are difficult in small children but should be included in their long-term follow-up as they grow and become mature enough to cooperate with such assessments. In fact, it is important to follow these children throughout primary and secondary school as many lesions, such as executing function, will not become apparent until school age.

Independently of the challenges of follow-up methods, we are far from having resolved the issue of cerebral lesions, which requires considerable research on how to optimize matters in many areas when treating such fragile patients. These areas include obstetric and fetal cardiology care and aiming at delivery at term, preoperative stabilization, timing of surgery, anesthetic protocols for best neuroprotection, conduct of cardiopulmonary bypass including the best mode of selective cerebral perfusion, and postoperative care including long-term issues such as support for the family and their social environment.

However, many of these issues may be settled only with randomized controlled trials, which take a long time and are limited by the fact that the patient population in most centers is relatively small and quite heterogeneous. These drawbacks may be partially neutralized using existing multicentric registries and international databases, but, even so, the challenge remains significant.

This challenge together with the whole history of cardiac surgery in general represents a good illustration of *the law of diminishing returns* that is well known to economists. This law has been phrased in various ways to adapt it to different circumstances, and we can do the same here: The more you progress, the harder it becomes to progress more, and the smaller the strides, eventually reaching a point where returns do not increase at all despite titanic efforts.

The *law of diminishing returns* reveals itself when the added efforts are directed at potentiating some of the elements of the issue at hand, but not all; the elements that have not changed become the limiting factors in the productivity of the elements that have been potentiated by the added efforts. Conversely, efforts directed at all elements, such that these are all potentiated uniformly in one mass, do increase the returns; the project at hand achieves a larger scale and its objectives become easier, faster and cheaper. This is the positive counterpart of *the law of diminishing returns* and is known as *the economy of scale,* although it is also sometimes facetiously called the law of *increasing* returns.

Our research into the issues of cerebral protection is hindered by *the law of diminishing returns* because, despite all our efforts, some elements of the work have not changed, namely the relatively small patient population in each center, their heterogeneity, and the fact that the length of follow-up needed is longer than the natural professional life of each investigator. Using existing multicentric registries and international databases, however, we can overcome these obstacles. In doing so, we replace *the law of diminishing returns* with *the economy of scale*. This, we believe, is the way forward.

## Data Availability

No datasets were generated or analysed during the current study.
